# Promotor Hypomethylation Mediated Upregulation of miR-23b-3p Targets PTEN to Promote Bronchial Epithelial-Mesenchymal Transition in Chronic Asthma

**DOI:** 10.3389/fimmu.2021.771216

**Published:** 2022-01-04

**Authors:** Yimin Guo, Xiaoqing Yuan, Luna Hong, Qiujie Wang, Shanying Liu, Zhaolin Li, Linjie Huang, Shanping Jiang, Jianting Shi

**Affiliations:** ^1^ Department of Respiratory Medicine, Sun Yat-Sen Memorial Hospital, Sun Yat-Sen University, Guangzhou, China; ^2^ Guangdong Provincial Key Laboratory of Malignant Tumor Epigenetics and Gene Regulation, Sun Yat-Sen Memorial Hospital, Sun Yat-Sen University, Guangzhou, China; ^3^ Institute of Pulmonary Diseases, Sun Yat-sen University, Guangzhou, China; ^4^ Department of Respiratory Medicine, Shaoxing People’s Hospital (Shaoxing Hospital, Zhejiang University School of Medicine), Shaoxing, China; ^5^ Breast Tumor Center, Sun Yat-Sen Memorial Hospital, Sun Yat-Sen University, Guangzhou, China; ^6^ Research Center of Medicine, Sun Yat-Sen Memorial Hospital, Sun Yat-Sen University, Guangzhou, China

**Keywords:** chronic asthma, miR-23b-3p, DNA hypomethylation, EMT, PTEN

## Abstract

Chronic asthma is characterized by airway inflammation and irreversible airway remodeling. Epithelial-mesenchymal transition (EMT) is a typical pathological change of airway remodeling. Our previous research demonstrated miR-23b inhibited airway smooth muscle proliferation while the function of miR-23b-3p has not been reported yet. Besides, miRNA is regulated by many factors, including DNA methylation. The function of miR-23b-3p and whether it is regulated by DNA methylation are worth exploring. Balb/c mice were given OVA sensitization to develop the asthmatic model. Expression of miR-23b-3p and EMT markers were measured by RT-qPCR, WB and immunohistochemistry (IHC). DNA methylation was detected by methylation-specific PCR (MSP) and the MassARRAY System. Asthmatic mice and TGF-β1-stimulated bronchial epithelial cells (BEAS-2B) showed EMT with increased miR-23b-3p. Overexpression of miR-23b-3p promoted EMT and migration, while inhibition of miR-23b-3p reversed these transitions. DNA methyltransferases were decreased in asthmatic mice. MSP and MassARRAY System detected the promotor of miR-23b showed DNA hypomethylation. DNA methyltransferase inhibitor 5’-AZA-CdZ increased the expression of miR-23b-3p. Meanwhile, PTEN was identified as a target gene of miR-23b-3p. Our results indicated that promotor hypomethylation mediated upregulation of miR-23b-3p targets PTEN to promote EMT in chronic asthma. miR-23b-3p and DNA methylation might be potential therapeutic targets for irreversible airway remodeling.

**Graphical Abstract:**
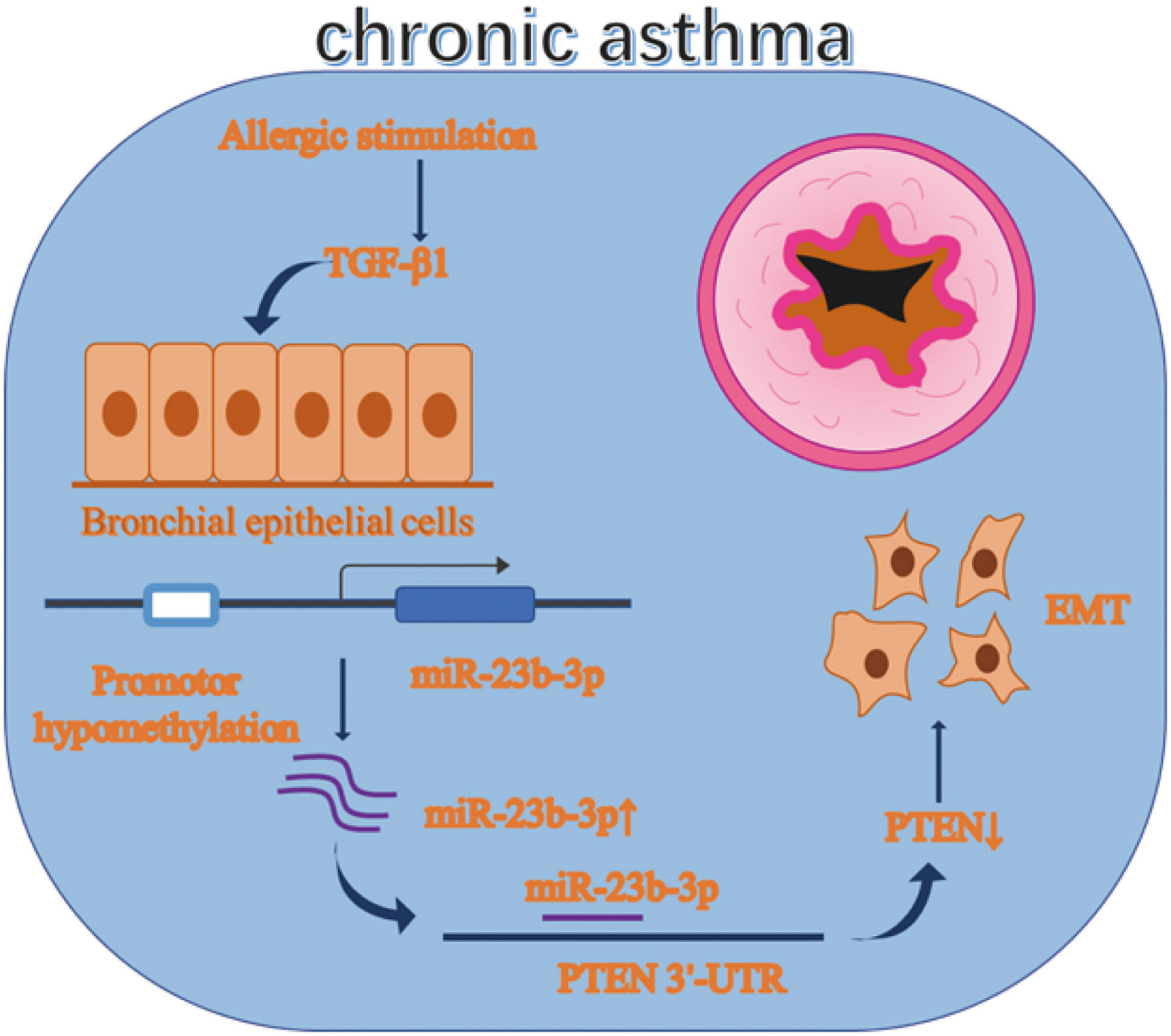


## Introduction

Chronic asthma is a severe global public health problem with increasing morbidity to 7.7 ± 0.02% and mortality to 10.5 ± 0.18/million (1). Patients with chronic asthma often suffer from dyspnea, cough, chest tightness, wheezing and other symptoms especially during midnight or early morning. Variable airflow obstruction and airway hyperresponsiveness (AHR) are typical changes of lung function. It greatly influences the quality of life of patients. Airway remodeling is the most typical lesion observed in chronic asthma which is characterized by epithelial-mesenchymal transformation (EMT), airway thickening, subepithelial fibrosis, airway smooth muscle cell hyperplasia, angiogenesis, and mucus over-secretion. Airway remodeling leads to irreversible loss of lung function. Current treatments can alleviate airway inflammation, but there are no effective treatments to prevent or reverse airway remodeling (2).

Allergens, viruses, and environmental pollutants stimulate airway epithelium to result in asthma. Airway epithelium, an important cell to control inflammation and immune response, expresses pattern recognition receptors, secretes endogenous risk signals, thereby activating innate and adaptive immunity (3). A typical pathological feature of asthmatic airway remodeling is repeated injury and repair, leading to EMT with epithelial barrier dysfunction (4). EMT shows decreased epithelial markers, increased mesenchymal markers, enhanced mobility, and extracellular matrix deposition, resulting in irreversible loss of lung function. Transforming growth factor β1 (TGF-β1) is a necessary factor that regulates lung organogenesis, homeostasis, and immune response (5, 6). Increased expression of TGF-β1 in lung tissues is considered a crucial factor in inducing the transformation of epithelial cells to mesenchymal cells in asthma, idiopathic pulmonary fibrosis (IPF), emphysema and cancer (7).

MicroRNAs (miRNA) are noncoding RNAs which are 19 to 25 nucleotides in size that inhibit gene expression *via* binding with the 3’-untranslated regions (3’-UTR) of the target gene mRNAs (8). miRNA play a crucial role in a variety of biological processes. In the past years, a great number of studies have also shown that miRNA plays a crucial influence in airway inflammation and airway smooth muscle cell proliferation in asthma (9, 10). Our previous studies found that miR-23b inhibited the cell proliferation of airway smooth muscle cells (11). The underlying role of miR-23b in the development of asthmatic airway remodeling, especially the remodeling of epithelial cells, deserves further studies. Additionally, the mechanism of miR-23b-3p acting through downstream target genes is also worth exploring.

PTEN is an important tumor suppressor, and it mainly plays a role in regulating the proliferation, energy metabolism and migration of tumor cells (12). It has been reported as a potential therapeutic target for asthma. Knocking down the PTEN gene induced airway remodeling and smooth muscle proliferation (13) whereas overexpression of PTEN inhibited smooth muscle proliferation in asthmatic mice (14). Whether PTEN plays a role in the epithelium is worth exploring.

Asthma is regulated by environmental factors and the immune system. The influence of environmental factors are partially regulated by epigenetic mechanisms, which include DNA methylation (15). In DNA methylation, a methyl group is transferred onto the C5 position to form 5-methylcytosine (5mC), hence regulating the transcription of the DNA sequence without affecting the genome sequence of DNA nucleotides (16, 17). Recent studies have focused on the entire DNA methylation or methylation of specific coding genes in asthma. However, research on non-coding RNA regulated by DNA methylation in asthma is scarce. The regulation of miRNA expression by DNA methylation has been reported in many diseases of cancer, but not in asthma (18, 19). For example, Tao et al. have demonstrated that methylation-mediated inhibition of miR-497 promoted the progression of breast cancer (20). Therefore, we conjectured that promoter methylation will influence the expression of miR-23b-3p and influence airway remodeling.

Here, our study proved that miR-23b-3p was increased in chronic asthma and it was regulated by DNA hypomethylation. Furthermore, PTEN was identified as a target gene of miR-23b-3p to regulate EMT in chronic asthma.

## Results

### Chronic Asthmatic Mice Showed EMT and Increased miR-23b-3p in Bronchial Epithelium

**Figure 1:**
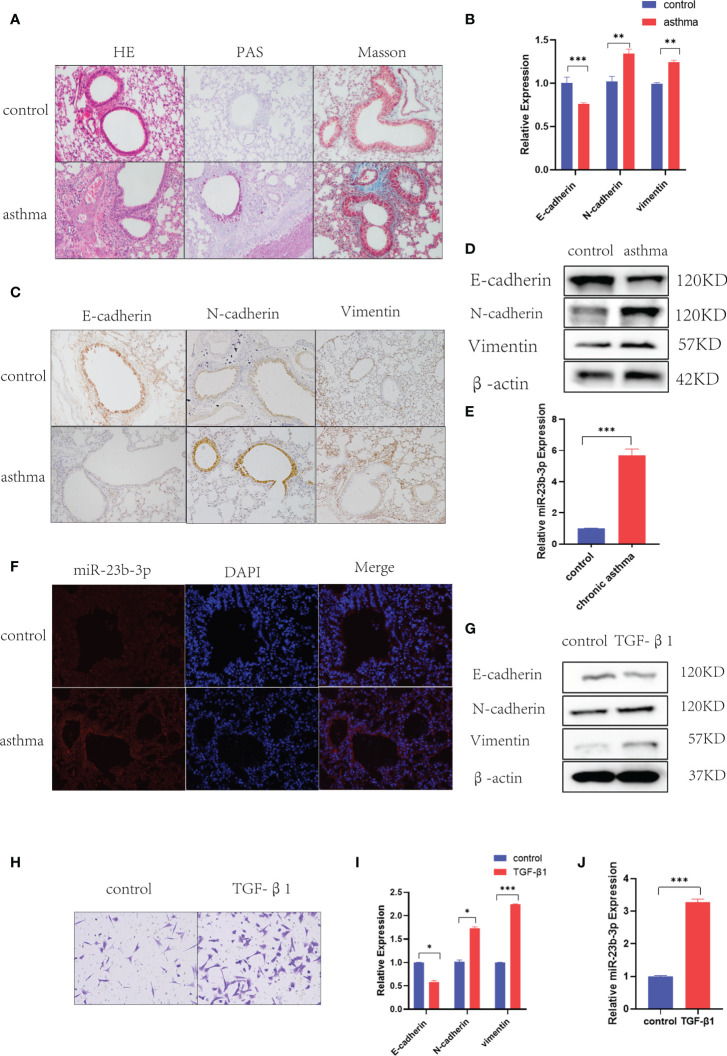
Chronic asthmatic mice showed EMT and increased miR-23b-3p in bronchial epithelium. **(A)** Representative images of HE, PAS and Masson staining of the lungs of mice. Representative photomicrographs (magnification 200×) are demonstrated for each subject. **(B)** RT-qPCR analysis of the mRNA levels of EMT markers E-cadherin, N-cadherin, and vimentin from the lungs of mice. **(C)** Immunohistochemical analysis of E-cadherin, N-cadherin, and vimentin in lung tissue and quantitative statistics of each groups. Representative photomicrographs (magnification 200×) are demonstrated for each subject. **(D)** Western blot analysis of the protein levels of EMT markers E-cadherin, vimentin, and N-cadherin from the lungs of mice. **(E)** RT-qPCR analysis of the mRNA levels of miR-23b-3p from the lungs of mice. **(F)** *In situ* hybridization analysis of miR-23b-3p location from the lungs of chronic asthmatic and control mice. Representative photomicrographs (magnification 200×) are demonstrated for each subject. **(G)** Western blot analysis of the protein levels of EMT markers E-cadherin, vimentin and N-cadherin from TGF-β1-stimulated BEAS-2B cells. **(H)** Representative images and quantitation of Transwell assay were performed in BEAS-2B cells with or without TGF-β1 treatment. Representative photomicrographs (magnification 100×) are demonstrated for each subject. **(I)** RT-qPCR analysis of the mRNA level of EMT markers E-cadherin, vimentin and N-cadherin from TGF-β1-stimulated BEAS-2B cells. **(J)** RT-qPCR analysis of the mRNA levels of miR-23b-3p from TGF-β1-stimulated BEAS-2B cells. The results are presented as the means ± SD (n=4~10). *P < 0.05, **P < 0.01, ***P < 0.001 compared with the indicated groups.

Histological analysis of lung tissues showed significant inflammatory cell infiltration, mucus over-secretion and airway remodeling in chronic asthma (**Figure 1A**). As EMT plays a vital role in airway remodeling, we detected the EMT markers E-cadherin, N-cadherin, and vimentin in the lung tissue of chronic asthmatic mice. RT-qPCR and Western blot indicated epithelial marker E-cadherin was decreased and mesenchymal markers N-cadherin and vimentin were increased in the lung tissue of chronic asthmatic-mice (**Figures 1B, D**). Immunohistochemical analysis further confirmed the existence of the EMT phenomenon in bronchial epithelial cells and para-bronchial cells in the lung tissue of chronic asthmatic mice (**Figure 1C** and **Figure S1B**). According to our previous research, miR-23b played an important role in airway remodeling. Hence, we measured the miR-23b-3p and miR-23b-5p in lung tissue. miR-23b-3p was significantly increased in the lungs of chronic asthmatic mice compare with the control group whereas miR-23b-5p was decreased (**Figure 1E** and **Figure S1C**). *In situ* hybridization showed that miR-23b-3p was increased in bronchial epithelial cells (**Figure 1F**). TGF-β1, an important cytokine, induced epithelial-mesenchymal transition and imitated the phenomenon of airway remodeling in chronic asthma. TGF-β1-stimulated BEAS-2B cells decreased the epithelial marker E-cadherin and increased the mesenchymal markers vimentin and N-cadherin (**Figures 1G, I**) and increased the migration (**Figure 1H**), which was another typical characteristic of EMT. The expression of miR-23b-3p was markedly increased in TGF-β1-stimulated BEAS-2B cells (**Figure 1J**).

### miR-23b-3p Promoted the EMT of Bronchial Epithelium

**Figure 2:**
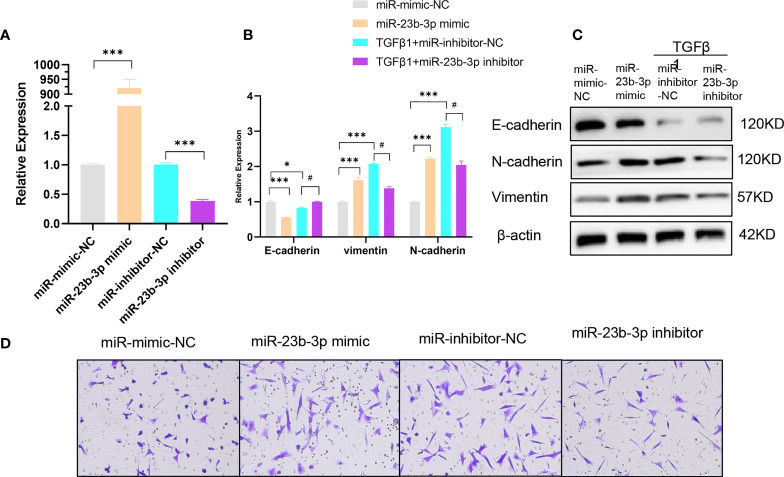
miR-23b-3p has a positive effect on EMT. **(A)** RT-qPCR analysis of mRNA levels of miR-23b-3p after transfection of miR-23b-3p mimic or miR-23b-3p inhibitor in BEAS-2B cells. **(B)** RT-qPCR analysis of mRNA levels of EMT markers E-cadherin, N-cadherin, and vimentin after transfection of miR-23b-3p mimic or miR-23b-3p inhibitor in BEAS-2B cells with or without TGF-β1 treatment. **(C)** Western blot analysis of protein levels of EMT markers E-cadherin, N-cadherin, and vimentin after transfection of miR-23b-3p mimic or miR-23b-3p inhibitor in BEAS-2B cells with or without TGF-β1 treatment. **(D)** Representative images and quantitation of Transwell assay after transfection of miR-23b-3p mimic or miR-23b-3p inhibitor in BEAS-2B cells with or without TGF-β1 treatment. Representative photomicrographs (magnification 100×) are demonstrated for each subject. The results are presented as the means ± SD of three independent experiments. *P < 0.05, ***P < 0.001 compared with the indicated groups. ^#^P < 0.05 compared with the indicated groups.

To further investigate the role of miR-23b-3p in EMT and migration of BEAS-2B cell, we transfected miR-23b-3p mimic or miR-23b-3p inhibitor into cells. miR-23b-3p was increased after transfection with miR-23b-3p mimic and decreased after transfection with miR-23b-3p inhibitor (**Figure 2A**). miR-23b-3p over-expression remarkably promoted EMT and migration. Down-regulation of miR-23b-3p inhibited EMT and migration (**Figures 2B–D**). However, miR-23b-3p did not influence the proliferation and apoptosis of BEAS-2B (**Figures S1D, E**).

### DNA Methyltransferase Expression Is Decreased and miR-23b-3p Was Regulated by Promotor Hypomethylation

**Figure 3:**
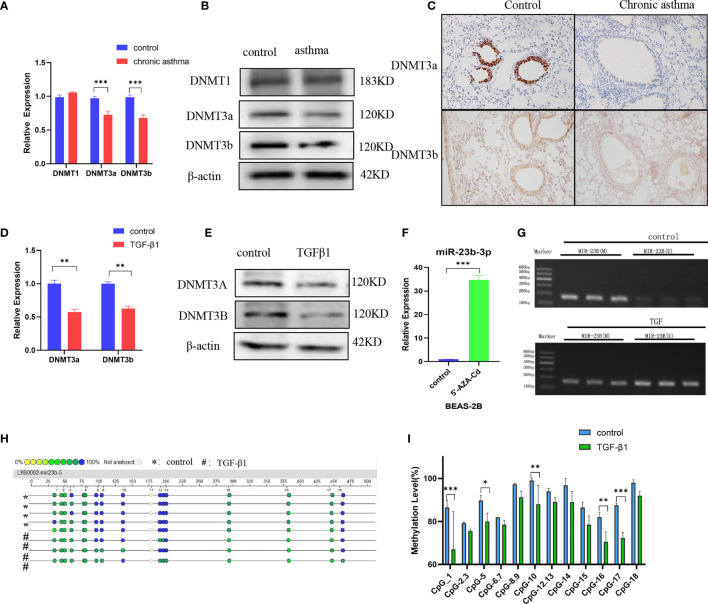
DNA methyltransferase expression is decreased and miR-23b-3p was regulated by promotor hypomethylation. **(A)** RT-qPCR analysis of the mRNA levels of DNA methyltransferases DNMT1, DNMT3a and DNMT3b from the lungs of mice. **(B)** Western blot analysis of the protein levels of DNMT3a and DNMT3b from the lung of mice. **(C)** Immunohistochemical analysis of DNMT3a and DNMT3b in lung tissue. Representative photomicrographs (magnification 200×) are demonstrated for each subject. **(D)** RT-qPCR analysis of the mRNA levels of DNA methyltransferase DNMT3a and DNMT3b from TGF-β1-stimulated BEAS-2B cells. **(E)** Western blot analysis of the protein levels of DNA methyltransferase DNMT3a and DNMT3b from TGF-β1-stimulated BEAS-2B cells. **(F)** RT-qPCR analysis of miR-23b-3p levels in 5’-AZA-CdR-treated BEAS-2B cells. **(G)** DNA methylation levels of the miR-23b-3p promotor region in TGFβ1-stimulated BEAS-2B cells as detected by MSP assay. **(H)** CpG sites methylation profile of the promotor of miR-23b-3p gene in TGFβ1-stimulated BEAS-2B cells as detected by MassARRAY. The different colors of each circles represent the percentage of methylation of each CpG site. **(I)** The percentage of methylation of each CpG site in miR-23b-3p promotor. The results are presented as the means ± SD (N=4~10). *P < 0.05, **P < 0.01, ***P < 0.001 compared with the indicated groups.

Asthma is an environmentally-related disease caused by epigenetic factors which include DNA methylation (21). The question was whether the expression of miR-23b-3p was regulated by DNA methylation. We measured the expression of DNA methyltransferase and found that the expression of DNMT3a and DNMT3b were significantly decreased in chronic asthma while DNMT1 did not have any statistical difference (**Figures 3A, B**). Immunohistochemical analysis confirmed that *de novo* methyltransferase DNMT3a and DNMT3b were significantly decreased in chronic asthma compared with the control group, and was especially expressed in bronchial epithelium (**Figure 3C**). DNMT3a and DNMT3b were also markedly decreased in TGF-β1-stimulated BEAS-2B cells (**Figures 3D, E**). We speculated that the expression of miR-23b-3p may be mediated by the decrease of DNA methyltransferases. We predicted the promoter region of the promoter of miR-23b-3p and found that the CpG islands were mainly located in the 1000 bp to 1500 bp area (**Figure S2**). BEAS-2B cells treated with 5’-AZA-CdZ, an inhibitor of DNA methyltransferases, significantly increased the expression of miR-23b-3p (**Figure 3F**), which indicated that the expression of miR-23b-3p was regulated by the promoter hypomethylation. Methylation-specific PCR assay demonstrated that TGF-β1 treatment decreased the DNA methylation and increased the DNA unmethylation of promotor region of miR-23b-3p (**Figure 3G**). The MassARRAY system detected each CpG site and CpG island methylation by high-throughput quantitative detection within 500 bp. The promotor of miR-23b-3p was located in a typical CpG unit, where the different colors of each circle represent the different levels of methylation. Yellow and green represented low methylation and blue represented full methylation. TGF-β1-treated groups decreased the methylation of miR-23b-3p promotor and CpG-1, CpG-5, CpG-10, CpG-16, CpG-17 clearly declined (**Figures 3H, I**). There was direct evidence that the upregulation of miR-23b-3p in BEAS-2B correlated with promotor hypomethylation.

### miR-23b-3p Promoted EMT *via* Silencing PTEN

**Figure 4:**
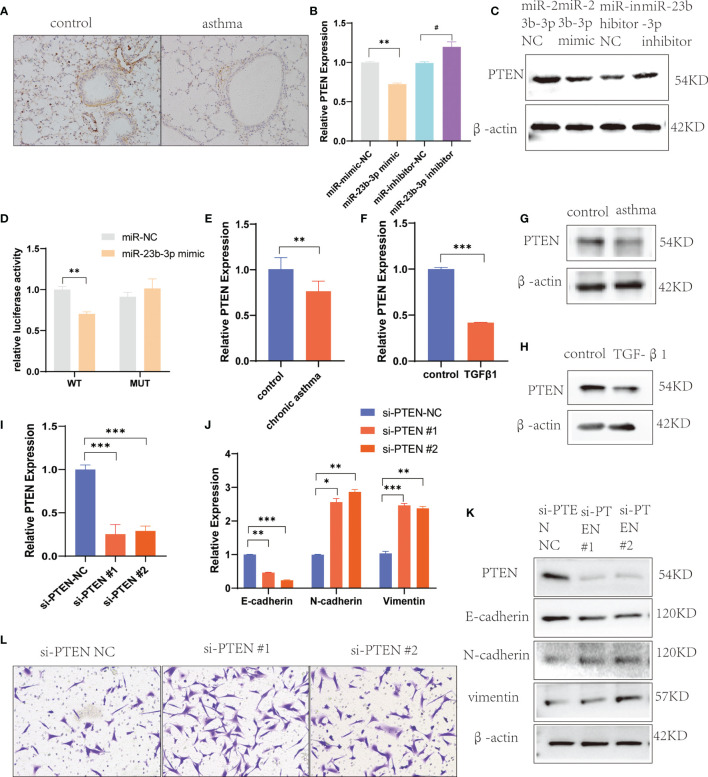
miR-23b-3p overexpression promote EMT *via* targeting PTEN gene. **(A)** Immunohistochemical analysis of PTEN in lung tissue. Representative photomicrographs (magnification 200×) are demonstrated for each subject. **(B)** RT-qPCR analysis of PTEN after transfection of miR-23b-3p mimic or miR-23b-3p inhibitor in BEAS-2B cells. **(C)** Western blot analysis of the protein levels of PTEN after transfection of miR-23b-3p mimic or miR-23b-3p inhibitor in BEAS-2B cells. **(D)** Dual-luciferase reporter assay. The relative luciferase activity was normalized to the Renilla luciferase activity assay after co-transfection with the miR-23b-3p mimic and miR-RB-REPORT constructs containing WT or MUT PTEN 3’-UTR region in 293T cell lines. **(E)** RT-qPCR analysis of the mRNA levels of PTEN in the lungs of chronic asthma and control mice. **(F)** RT-qPCR analysis of the mRNA levels of PTEN in TGF-β1-treated BEAS-2B cells. **(G)** Western blot analysis of the protein levels of PTEN in the lungs of chronic asthma and control mice. **(H)** Western blot analysis of the protein levels of PTEN in TGF-β1-treated BEAS-2B cells. **(I)** RT-qPCR analysis of the mRNA levels of PTEN in BEAS-2B cells silencing the PTEN gene. **(J)** RT-qPCR analysis of the mRNA levels of EMT markers in BEAS-2B cells silencing the PTEN gene. **(K)** Western blot analysis of the protein levels of PTEN and EMT markers in BEAS-2B cells silencing the PTEN gene. **(L)** Representative images and quantitation of Transwell assay in BEAS-2B cells silencing the PTEN gene. Representative photomicrographs (magnification 100×) are demonstrated for each subject. The results are presented as the means ± SD of three independent experiments. *P < 0.05, **P < 0.01, ***P < 0.001 compared with the indicated groups. ^#^P < 0.05 compared with the indicated groups.

To explore the mechanism by which miR-23b-3p regulated EMT, we searched for potential downstream regulatory targets of miR-23b-3p using several bioinformatic databases, including miRDB, miRBase, Targetscan and PubMed, and found that the 3’-UTR of PTEN mRNA contained sequences that were potential targets of miR-23b-3p (**Figure S3A**). The expression of PTEN was down-regulated in BEAS-2B cells transfected with miR-23b-3p mimic, while miR-23b-3p inhibitor increased the expression of PTEN (**Figures 4B, C**). We built the plasmid containing the binding sequence of miR-23b-3p and PTEN or mutated binding sequence with luciferase reporter structure for the dual luciferase reporter assay (**Figure S3B**). It revealed the regulation of PTEN by miR-23b-3p. The miR-23b-3p mimic significantly suppressed the luciferase activity of WT-PTEN-3’UTR, while it had no inhibitory effect on the luciferase activity of MUT-PTEN-3’UTR (**Figure 4D**). Moreover, we tested the PTEN expression in chronic asthmatic mice and TGF-β1-treated BEAS-2B cells and found that PTEN was decreased in the lung tissue of chronic asthmatic mice (**Figures 4A, E, G**) and TGF-β1-treated cells (**Figures 4F, H**). To further verify the function of PTEN in EMT, we silenced the expression of PTEN by transfecting siRNA in BEAS-2B cells and found that E-cadherin was decreased and the expression of vimentin and N-cadherin was increased (**Figures 4I–K**). Down-regulation of PTEN also increased the migration of BEAS-2B cells (**Figure 4L**). Taken together, these results proved that PTEN was a downstream target of miR-23b-3p which induced EMT and airway remodeling.

### Downregulation of miR-23b-3p Reversed EMT and Airway Remodeling *In Vivo*

**Figure 5:**
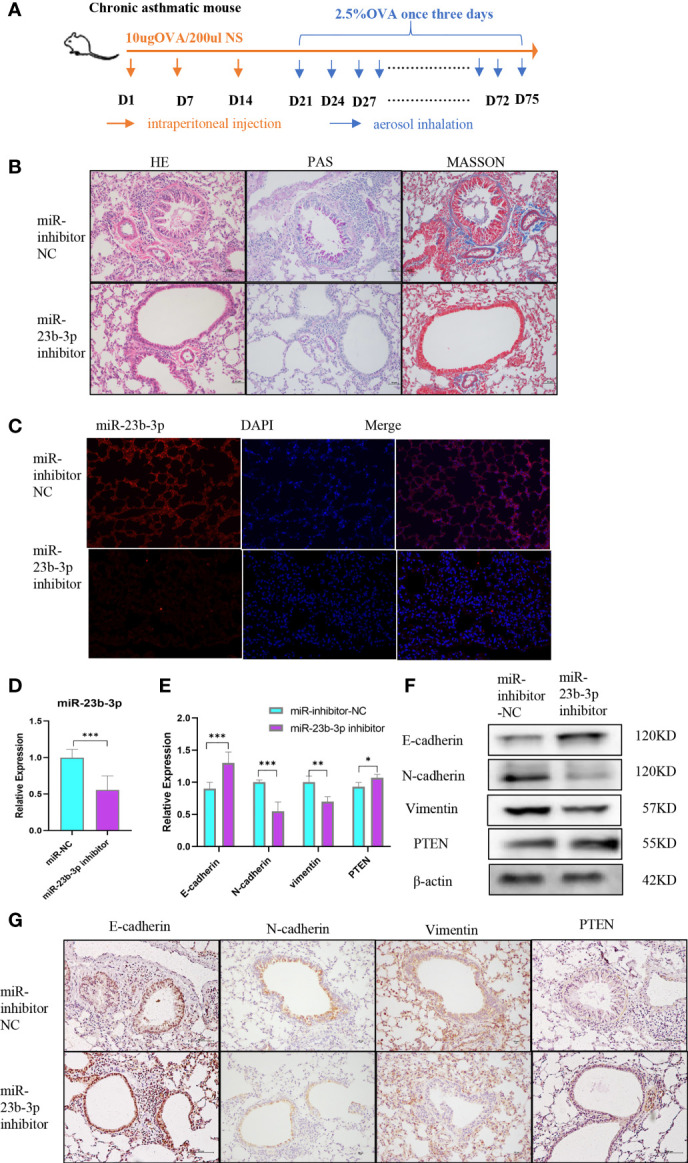
Downregulation of miR-23b-3p inhibited EMT and airway remodeling *in vivo*. **(A)** Experimental design of ovalbumin induced chronic asthma and AAV treatment. **(B)** Representative images of HE, PAS and Masson staining of the lungs of miR-23b-3p inhibitor-treated asthmatic mice and control mice. Representative photomicrographs (magnification 200×) are demonstrated for each subject. **(C)** *In situ* hybridization analysis of miR-23b-3p from the lungs of miR-23b-3p inhibitor-treated asthmatic mice and control mice. Representative photomicrographs (magnification 200×) are demonstrated for each subject. **(D)** RT-qPCR analysis of the mRNA levels of miR-23b-3p and EMT markers from the lungs of miR-23b-3p inhibitor-treated asthmatic mice and control mice. **(E)** RT-qPCR analysis of the mRNA levels of PTEN and EMT markers from the lungs of miR-23b-3p inhibitor-treated asthmatic mice and control mice. **(F)** Western blot analysis of the protein levels of EMT markers and PTEN gene from the lungs of miR-23b-3p inhibitor-treated asthmatic mice and control mice. **(G)** Immunohistochemical analysis of PTEN and EMT markers from the lungs of miR-23b-3p inhibitor-treated asthmatic mice and control mice. Representative photomicrographs (magnification 200×) are demonstrated for each subject. The results are presented as the means ± SD (n=4~10). *P < 0.05, **P < 0.01, ***P < 0.001 compared with the indicated groups.

Prior to the establishment of a chronic asthma model, AAV containing miR-23b-3p inhibitor was administered *via* tracheal drip into the airway (**Figure 5A**). HE, PAS and Masson staining of lung tissues showed that miR-23b-3p inhibitor alleviated airway inflammatory cell infiltration, mucus over-secretion, and especially airway remodeling (**Figure 5B**). The expression of miR-23b-3p in the lung were significantly decreased (**Figures 5C, D**). Besides, we found the expression of epithelial marker E-cadherin and the target gene PTEN were increased, and mesenchymal markers N-cadherin and vimentin were decreased in the lung tissue of mice treated with miR-23b-3p inhibitor compared with the control group (**Figures 5E–G**). Downregulation of miR-23b-3p inhibited EMT and airway remodeling *in vivo* which indicated that miR-23b-3p may be a potential therapeutic target of airway remodeling.

## Discussion

Asthma is a severe public disease characterized by airway inflammation and airway remodeling accompanied by irreversible decline of lung function. In the present study, we discovered that the expression of miR-23b-3p was increased and DNA methyltransferases were decreased in the lung tissue of asthmatic mice. The expression of miR-23b-3p was regulated by hypomethylation of the promotor region of miR-23b. Furthermore, we identified PTEN as an important downstream target for miR-23b-3p, and miR-23b-3p overexpression promoted EMT *in vitro* and *in vivo*. In all, the expression of miR-23b-3p was regulated by DNA methylation and miR-23b-3p may act as a potential therapeutic target for airway remodeling in chronic asthma.

We built the chronic asthmatic mice model and found that the lung tissue showed EMT, accompanied by a significant increase of miR-23b-3p. Overexpression of miR-23b-3p promoted EMT in airway epithelium, while inhibition of miR-23b-3p inhibited EMT and airway remodeling *in vitro* and *in vivo*. In addition, our previous study showed that miR-23b inhibited the proliferation of airway smooth muscle in mice with chronic asthma. We detected the expression of miR-23b-3p and miR-23b-5p. miR-23b-5p was decreased, which may be the main factor affecting smooth muscle proliferation. miR-23b-3p is significantly elevated in the lung tissue of chronic asthma, and its function is intriguing. miR-23b-3p may play a more important role in other cells, such as epithelial cells. FISH analysis results confirmed that miR-23b-3p was expressed in epithelial cells, and its function may be related to EMT. Recent studies have found that miRNA plays an important role in many diseases, including tumors, inflammation, and immune diseases. Research on miR-23b-3p mainly focused on tumors, aneurysms, primary nephrotic syndromes, and other diseases (22–25). Yunpeng Sun et al. and Tao Yang et al. have confirmed that miR-23b-3p causes EMT in liver cancer (26, 27). Wang et al. reported that miR-23b promote tumor cell proliferation, migration and EMT by targeting E-cadherin in nasopharyngeal carcinoma (28). Majid et al. reported that miR-23b promote EMT through targeted Src pathway in prostate cancer (29). These two articles did not explore the function of miR-23b-3p and miR-23b-5p. Our analysis confirmed that PTEN gene was the target gene of miR-23b-3p, but cannot exclude the direct binding of miR-23b-3p and E-cadherin. However, these have little influence on the conclusion of our study. The function of miR-23b-3p in asthma has yet to be elucidated. Airway epithelial junction forms and maintains epithelial barrier besides regulating innate and adaptive immune response by releasing inflammatory factors and chemokines (30). EMT is one of the most important pathological manifestations of airway remodeling, which leads to the impairment of epithelial barrier function, ECM deposition, and irreversible decline of lung function (30). Our results indicated that miR-23b-3p inhibited the EMT of bronchial epithelium *in vivo* and *in vitro*, which may be a clue to ameliorate airway remodeling of asthma.

pri-miR-23b is produced in the nucleus and transferred to the cytoplasm for modification and the production of pre-miR-23b. pre-miR-23b is processed to miR-23b-3p and miR-23b-5p by nuclease. miRNA expression is regulated by many factors, including target gene, DNA methylation, RNA methylation, histone modification, and other non-coding RNA (31–33). DNA methylation is one of the epigenetic mechanisms that mediated the reversible modification of DNA, affected gene expression without changing the genome sequence, and participated in the pathogenesis of COPD, pulmonary hypertension, and asthma (34). To confirm that miR-23b-3p is regulated by promoter hypomethylation, we queried the sequence of miR-23b-3p, which belongs to miR-23b/27b/24 cluster and located in the c9orf3 host gene (35). The c9orf3 host gene (ENST00000297979) where the miR-23b/27b/24 cluster is located contains 15 extron gene (36). The length of c9orf3 host gene is 764107 bp, far exceeding the length of the miR-23b/27b/24 cluster. Nishida K et al. and Chi Lam Au Yeung et al. reported that the expression levels of the miR-23b/27b/24 cluster regulated through DNA methylation of the host gene C9orf3 (36, 37). The promoter region we detected is in the upstream DNA sequence corresponding to miR-23b-3p, which regulates the production of miR-23b-3p. Furthermore, we treated BEAS-2B cells with DNA methyltransferase inhibitor 5’-aza-cdz, and the expression of miR-23b-3p increased significantly. Therefore, we draw a conclusion that promoter hypomethylation regulates the up-regulation of miR-23b-3p. Besides, no literature has confirmed that C9orf3 gene is involved in the pathogenesis of asthma and the EMT of bronchial epithelial cells. It is very innovative to explore the regulatory role of C9orf3 gene in asthma.A meta-analysis of epigenetic DNA methylation of 4-16 year-old asthmatic children showed that methylation levels were low at all relevant sites in asthmatic children (21). A genome-wide study on DNA methylation in the nasal epithelium of asthmatic children revealed 8664 differentially methylated genes were mainly related to epithelial barrier function, epithelial integrity, and immune regulation, which further confirmed that DNA methylation plays a major role in airway epithelium (38). The methylation levels of promoter regions of key genes, such as IL-4, IFN-γ, IL-33, CCL26 and MAPK1 in airway epithelium in asthmatic subjects were significantly decreased compared with normal subjects (39–41). At present, the main research focused on comparing the methylation level of whole genomes or some important coding genes in asthma with non-asthma. However, the regulatory effects of DNA methylation on non-coding RNA, including miRNA, has not been elucidated in asthma. Only Zhao et al. reported that miR-126 was overexpressed in the exosomes of the serum of asthmatic patients, while DNMT1 expression was decreased, however the regulatory mechanism was not elucidated (42). Our study found that miR-23b-3p was elevated and DNA methyltransferases were decreased in chronic asthma. Additionally, hypomethylation of the promotor region of miR-23b induced the increased expression of miR-23b-3p, which resulted in EMT and airway remodeling of chronic asthma. Moreover, Zhao et al. found a significant decrease of DNMT1 in the serum of patients, but he did not detect DNMT3a and DNMT3b (42). Our study found that DNMT3a and DNMT3b were significantly decreased in the lung tissue, especially in the airway epithelium of asthmatic mice, while DNMT1 did not change. It was concluded that DNMT3a and DNMT3b mediated *de novo* methylation of miR-23b promotor, which plays a vital role in EMT and airway remodeling in asthma.

miRNAs were short-chain non-coding RNAs which inhibited the expression of mRNA by targeting the 3’-UTR of target mRNA. We identified PTEN as the target gene of miR-23b-3p through bioinformatics analysis and literature search. PTEN is an important tumor suppressor gene which mainly focused on regulating the proliferation, energy metabolism and migration of tumor cells (31). In our study, PTEN was detected as the target gene of miR-23b-3p. Overexpression of miR-23b-3p inhibited the expression of PTEN and promoted EMT. Meanwhile, silencing PTEN expression induced EMT. We also verified the function of miR-23b-3p inhibitor *in vivo* and *in vitro* and found that the expression of PTEN was significantly increased. Xin Wen et al. also found PTEN to be a potential therapeutic target. Knockdown of the PTEN gene induced airway remodeling and smooth muscle proliferation in asthma (13). Resveratrol and epigallocatechin-3-gallate played an anti-asthmatic role by regulating the PTEN/PI3K/Akt pathway (14, 43)

In conclusion, our study confirmed that 1) Overexpression of miR-23b-3p induced epithelial mesenchymal transition and airway remodeling in asthmatic mice. 2) Reduced DNMT3a and DNMT3b expression induced demethylation of miR-23b promoter regions, resulting in upregulation of miR-23b-3p. 3) PTEN, a target gene of miR-23b-3p, participated in the process of EMT. In all, the expression of *de novo* methyltransferases DNMT3a and DNMT3b were decreased, leading to demethylation of the promoter region of miR-23b, thereby upregulating the expression of miR-23b-3p. High expression of miR-23b-3p induced airway epithelial-mesenchymal transition and airway remodeling by targeting PTEN. DNA *de novo* methylation and miR-23b-3p may be potential therapeutic targets for airway remodeling in chronic asthma.

## Material and Methods

### Animal Experimental Protocols

Six-to-eight-week old male Balb/c mice were bought from the Sun Yat-sen University Animal Experiment Center. 40 mice were equally divided into four groups by random, including control group, chronic asthma group, miR-NC group and miR inhibitor group. Adeno-associated virus (AAV) (GeneChem, China), which contained the expression of miR-NC and miR-23b-3p inhibitor, were respectively administered to the miR-NC group and miR inhibitor group by tracheal drip on day 0. After AAV treatment, mice were given OVA stimulation and were challenged to build the chronic asthma model. The chronic asthmatic mouse model was established by using ovalbumin (OVA) stimulation and challenge. The mice were stimulated with intraperitoneal injection of 10 µg of OVA emulsified in 1 mg of aluminum hydroxide (Al(OH)_3_) in a total volume of 0.2 mL PBS on days 0, 7, 14, and 21, and was then exposed to 2.5% OVA aerosol using an air atomizer every three days. Normal saline was used as a control. Mice were euthanized after 20 times of atomization on day 78. The experimental protocol was approved by the Animal Ethics Committee of Sun Yat-sen University (No. 2020000197).

### Cell Culture

Human bronchial epithelial cells (BEAS-2B) (Fuheng Biology, China) were cultivated in 4.5 g glucose DMEM medium (Gibco, USA), with 10% fetal bovine serum (FBS) (Sigma, USA), 100 IU/mL of penicillin and streptomycin (HyClone, USA) in incubators containing 5% CO_2_ at 37°C. BEAS-2B were treated with 10 µg/mL TGF-β1 (Proteintech, USA). RNA samples were extract after 24 hr of TGF-β1 treatment and protein samples were extracted after 48 hr of treatment.

### Cellular Transfection

BEAS-2B were transfected with miR-NC, miR-23b-3p mimic, miR-inhibitor-NC, miR-23b-3p inhibitor, siRNA-NC, si-PTEN#1, and si-PTEN#2 (Synbio Tech, China) by using Roche Transfection Reagent (Thermo Fisher Scientific, USA) according to the manufacturer’s protocols.

### RT-qPCR

Total RNA was extract using TRIzol (TAKARA, Japan). The NanoDrop One Spectrophotometer was used to measure the purity and concentration of RNA. 500 ng of RNA was used for the reverse transcription (RT) reactions, and quantitative polymerase chain reaction (RT-qPCR) was performed with LightCycler FastStart DNA master SYBR green I (TAKARA, Japan) fluorescent dye using Roche 480 PCR machine (Roche, USA). GAPDH and U6 were used as controls. Primers sequences were available in **Supplementary Table 1**.

### Western Blot

Total protein was extracted using RIPA lysis buffer (Epizyme, China) and quantified using BCA protein quantification kit (Epizyme, China) according to the manufacturer’s protocol. The protein was suspended in 5×loading buffer (Epizyme, China), boiled at 98°C for 10 min and stored at -80°C. The protein sample was electrophoresed on 10% electrophoresis gels at 120 mV for 1 hr, and transferred to polyvinylidene difluoride (PVDF) membranes (Millipore, USA) *via* electroblotting at 250 mA for 90 min. The membranes were blocked with 5% non-fat powdered milk in 0.1% TBST solution at room temperature for 1 hr, followed by overnight incubation at 4°C with the primary antibodies, including E-cadherin 1:1000 (St Johns, UK) N-cadherin 1:1000 (Servicebio, China), vimentin 1:1000 (Servicebio, China), PTEN 1:1000 (CST, USA), DNMT1 1:1000 (Abcam, UK), DNMT3a 1:1000 (Abcam, UK), DNMT3b 1:1000 (Abcam, UK), GAPDH 1:1000 (CST, USA), and β-actin 1:1000 (Servicebio, China). HRP-labeled IgG (Beyotime, China) was used as the secondary antibody and was exposed with chemiluminescent substrate reagent (Epizyme, China).

### Proliferation Assay

BEAS-2B were seeded into each well of a 96-well plate (5000 per well), and the proliferation ability was determined by CCK8 test. CCK8 solution was added (10 µl/well) to each well and incubated at 37°C for 2 hr. The OD value of each sample was measured using a microplate reader at 450 nm.

### Migration Assay

Migration assay was performed using 8 µm Transwell chambers (8 µM pore size, Corning, USA). Cells were treated with trypsin and re-suspended in serum-free medium. 200 µL cell suspension containing 2×10^4 cells were added to the Transwell chamber. 2% FBS medium with or without TGF-β1 treatment was added to the wells under the chambers. The chambers were fixed with 4% glutaraldehyde solution after 12 hr to determine the number of migratory cells. The lower surfaces of the chambers were stained with crystal violet dye for 20 min. The stained chambers were viewed using light microscopy.

### Immunohistochemistry (IHC)

The expression of E-cadherin, vimentin, N-cadherin and PTEN were stained using IHC in the lung tissue. After deparaffinization and hydration using xylene and sequentially decreasing concentrations of ethanol, the tissues were incubated in 0.01 mol/L tris-EDTA buffer (PH 6.0) (Servicebio, China) in a high-temperature and high-pressure container for 10 min for antigen retrieval. Then, the tissues were cooled and incubated in 3% H_2_O_2_ for 10 min to inactivate endogenous peroxidases and blocking was performed with normal goat serum for 30 min. The tissues were incubated with primary antibodies E-cadherin (1:500) (St John’s, UK), vimentin (1:200) (Servicebio, China), N-cadherin (1:200) (Servicebio, China), and PTEN (1:100) (CST, USA) overnight at 4°C, followed by HRP-labelled secondary antibody for 37°C for 30 min and DAB staining kit according to the manufacturer’s protocol (SolelyBio, China). The stained tissues were viewed using light microscopy.

### *In Situ* Hybridization of miR-23b-3p

The mmu-miR-23b-3p probe (5’-GTGGTAATCCCTGGCAATGTGAT-3’) was tagged with 3’ and 5’ digoxigenin and modified with LNA nucleotides (Servicebio, Wuhan, China). The probe-target complex was detected using an anti-digoxigenin-alkaline phosphate conjugate, DAB as the chromogen. The tissues were dewaxed and dehydrating using xylene and sequentially decreasing concentrations of ethanol. Tissues were incubated in repairing fluid in a high-temperature and high-pressure container for 10 min for antigen retrieval. Tissues were incubated with proteinase K under 37°C for 20 min and washed with PBS three times for 5 min each. The tissues were then cooled and incubated in 3% H_2_O_2_ for 10 min to inactivate endogenous peroxidases, and were blocked with pre-hybridization fluid under 60°C for 1 hr. The probe was added onto tissues at 4°C overnight. Then, the tissues were briefly washed in pre-warmed 5×, 1× and 0.5× SSC (37°C) three times for 5 min each. The tissues were incubated with the anti-digoxigenin secondary antibody at 37°C for 50 min and DAB staining kit according to the manufacturer’s protocol. The stained tissues were viewed using light microscopy. All procedures were performed under RNase-free condition.

### Dual-Luciferase Reporter Assay

Bioinformatics analysis was performed using the following programs: Targetscan, miRBase, miRDB and PubMed. We speculate PTEN as the potential target of miR-23b-3p correlated with EMT. The WT-PTEN plasmids contained the 3’-untranslated regions (3’-UTR) of human PTEN genomic DNA binding site and firefly fluorescent plasmid structure. The MUT-PTEN plasmids contained the mutated 3’-untranslated regions (3’-UTR) of human PTEN genomic DNA binding site and firefly fluorescent plasmid structure. 293T cells were co-transfected with Renilla fluorescent plasmid and WT-PTEN plasmid or MUT-PTEN plasmid, and were transfected with miR-NC or miR-23b-3p mimic for 48 hr. Dual-luciferase reporter assay kit (Promega, USA) was used to measure the luciferase according to the manufacturer’s protocol.

### Methylation Specific PCR

Total DNA was extracted using the DNA extraction kit (Tiangen, China). NanoDrop One Spectrophotometer was used to measure the purity and concentration of DNA. 500 ng DNA was treated with bisulphite by using the DNA bisulphite kit (Tiangen, China) according to the manufacturer’s protocol. Specific methylation PCR kit (Tiangen, China) was used for specific amplification of Bisulfited DNA of the promotor region of miR-23b-3p by using the Biometra TG96 PCR machine (Bio-rad, USA). The amplified products were detected by DNA electrophoresis. Bisulfited DNA were detected by MassARRAY System (BGI, China) to verify the methylation of each CpG island.

### Statistical Analysis

Statistical analysis was performed using GraphPad 8.0 software. Student’s *t*-test, one-way ANOVA and two-way ANOVA were used to determine the differences among different groups. A *p*-value < 0.05 was considered as statistical significance. All data were expressed as the means ± standard deviation (M ± SD).

## Data Availability Statement

The raw data supporting the conclusions of this article will be made available by the authors, without undue reservation.

## Ethics Statement

The animal study was reviewed and approved by Animal Ethics Committee of Sun Yat-sen University.

## Author Contributions

YG and XY designed experimental scheme, implemented research process, designed the article framework, and wrote the article. LNH and QW collected the original data and carried out the statistical analysis. SL implemented research process and collected original data. ZL and LJH provided the technical support. SJ and JS obtained the research funding, designed experimental scheme, and proposed research options.

## Funding

This study was supported by National Nature Science Foundation of China (No.81670022, 81803636, 81700033 and 82071804), Nature Science Foundation of Guangdong Province (No. 2017A030313822 and 2018A0303130329), Guangzhou Science and Technology Program Key Project (No 201704020123, 202008040003 and No. 202102020429), the Emergency Program for Guangzhou Regenerative Medicine and Health Guangdong Laboratory of China (2020GZR110106003).

## Conflict of Interest

The authors declare that the research was conducted in the absence of any commercial or financial relationships that could be construed as a potential conflict of interest.

## Publisher’s Note

All claims expressed in this article are solely those of the authors and do not necessarily represent those of their affiliated organizations, or those of the publisher, the editors and the reviewers. Any product that may be evaluated in this article, or claim that may be made by its manufacturer, is not guaranteed or endorsed by the publisher.

## Abbreviations

AAV, adeno-associated virus; AHR, airway hyper reactivity; AKT(PKB), protein kinase B; AL(OH)3, aluminum hydroxide; BCA, bicinchoninic acid; BEAS-2B, human bronchial epithelial cells; COPD, chronic obstructive pulmonary diseases; DMEM, dulbecco’s modified eagle medium; DNMT, DNA methyltransferase; EMT, epithelial-mesenchymal transition; FBS, fetal Bovine Serum; FISH, fluorescence *in situ* hybridization; GAPDH, glyceraldehyde-3-phosphate dehydrogenase; HE, hematoxylin-eosin staining; IHC, immunohistochemistry; IL, interleukin; IPF, idiopathic pulmonary fibrosis; MAPK, mitogen-activated protein kinase; MIRNA, microRNA; MSP, methylation-specific PCR; OVA, ovalbumin; PAS, periodic Acid-Schiff staining; PBS, phosphate Buffered Saline; PI3K, phosphatidylinositol kinase 3; PTEN, phosphatase and tensin homolog deleted on chromosome ten; RT-qPCR, Real-time Quantitative polymerase chain reaction; TGF-β1, transforming growth factor-β1; UTR, untranslated regions; WB, western blot.
