# Supplementary material for: Promotor Hypomethylation Mediated Upregulation of miR-23b-3p Targets PTEN to Promote Bronchial Epithelial-Mesenchymal Transition in Chronic Asthma
Source: Front Immunol. 2022 Jan 4;12:771216. doi: 10.3389/fimmu.2021.771216 (PMC8765721; doi:10.3389/fimmu.2021.771216)
Supplement: Supplementary file 1 [file DataSheet_1.pdf]

Supplementary Figure 1

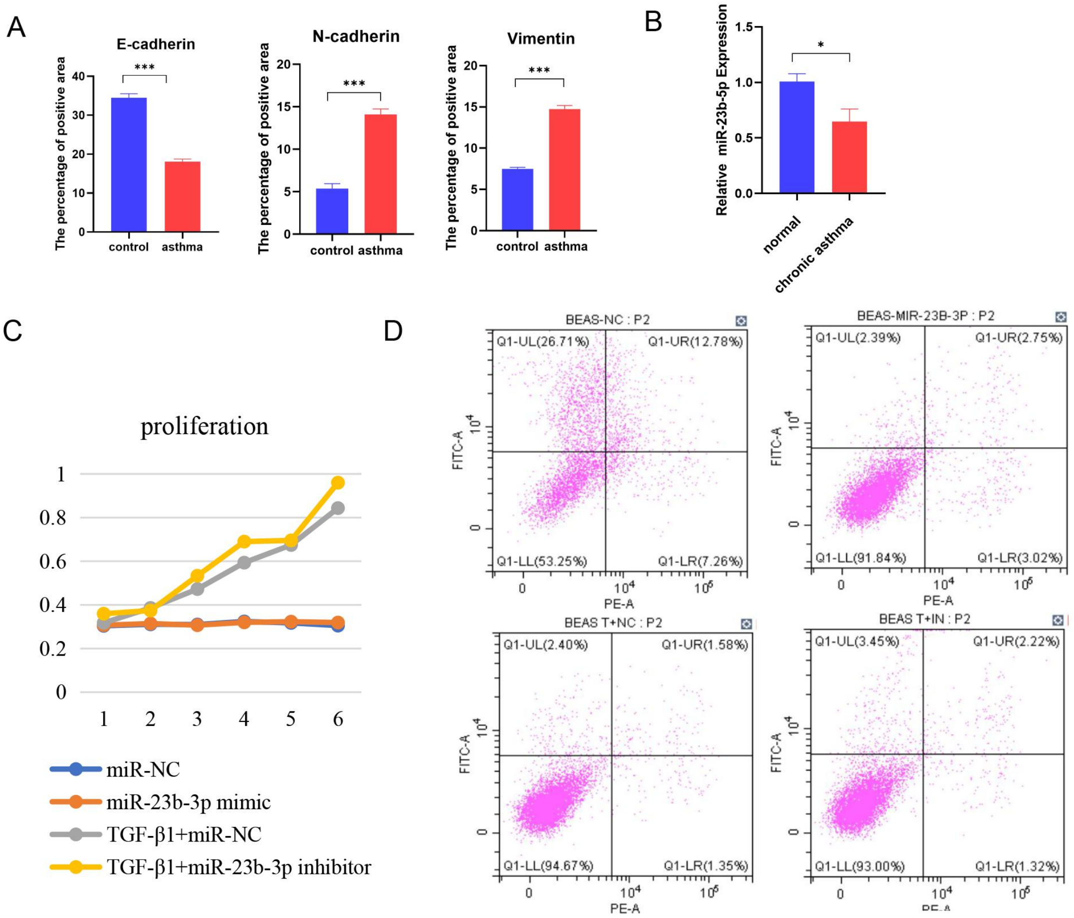

Supplementary figure 1: (A) Immunohistochemical analysis of E-cadherin, N-cadherin, and vimentin in lung tissues and quantitative statistics of each group. (B) RT-qPCR analysis of the mRNA level of miR-23b-5p from the lung of mice. (C) CCK8 test of BEAS-2B transfected with miR-23b-3p mimic or miR-23b-3p inhibitor with or without TGF-β1 treatment in 5 days. (D) Flow cytometry analysis of apoptosis of BEAS-2B transfected with miR-23b-3p mimic or miR-23b-3p inhibitor with or without TGF-β1 treatment. The results are presented as the means ± SD of three independent experiments. *P<0.05, **P<0.01, ***P<0.001 compared with the indicated groups.

Supplementary Figure 2

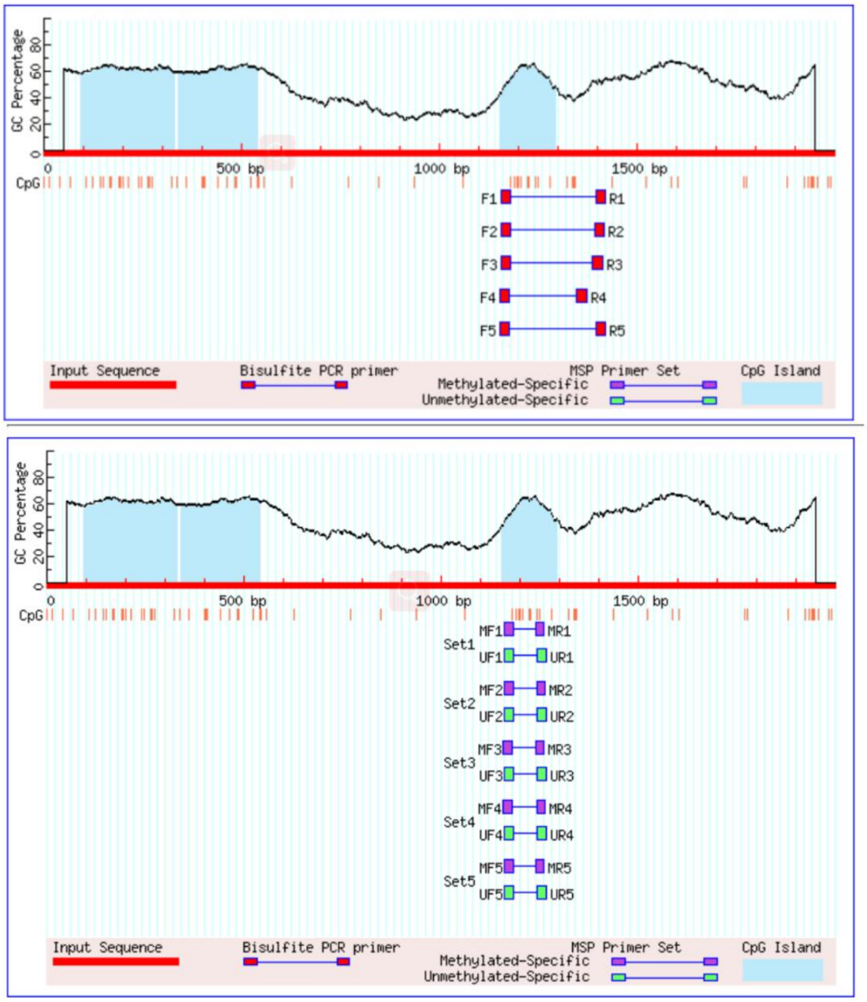

Supplementary figure 2: Genomic structure and distribution of miR-23b-3p CpG dinucleotides over the transcription start site (TSS).

Supplementary Figure 3

A

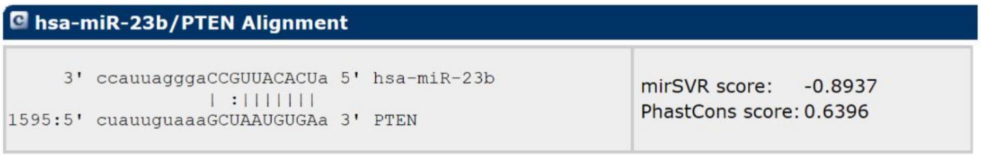

>hsa-miR-23b-3p MIMAT0000418
AUCACAUUGCCAGGGAUUACCAC
miRNA SEQ
5'---AUCACAUUGCCAGGGAUUACCAC---3'
3'---TAGTGTAACGGTCCCTAATGGTG---5'

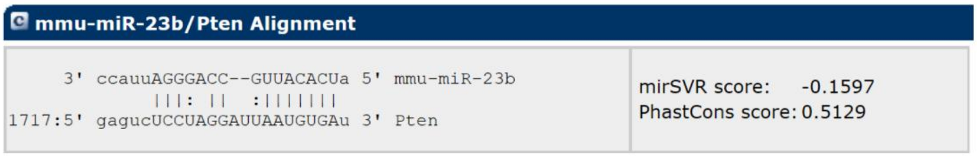

>mmu-miR-23b-3p MIMAT0000125
AUCACAUUGCCAGGGAUUACC
miRNA SEQ
5'---AUCACAUUGCCAGGGAUUACC---3'
3'---TAGTGTAACGGTCCCTAATGG---5'

B

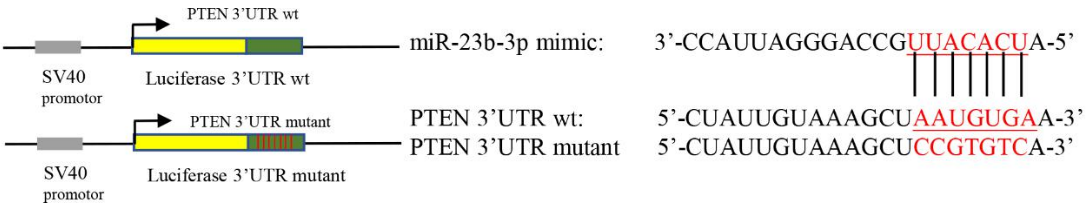

Supplementary figure 3: (A) mmu-miR-23b-3p and has-miR-23b-3p were both directly targeted PTEN with same binding site. (B) Dual-luciferase reporter assay. The relative luciferase activity was normalized to the renilla luciferase activity assay after cotransfection with the miR-23b-3p mimic and miR-RB-REPORT construts containing WT or MUT PTEN 3'UTR region in 293T cell lines.

Supplementary Table 1: Primer sequences applied for gene expression analyses

species	gene	Forward primer	Reversed primer
human	E-cadherin	ATTTTTCCCTCGACACCCGAT	TCCCAGGCGTAGACCAAGA
	N-cadherin	TCAGGCGTCTGTAGAGGCTT	ATGCACATCCTTCGATAAGACTG
	Vimentin	AGTCCACTGTACCGGAGAC	CATTTCACGCATCTGGCGTTC
	β-actin	CATGTACGTTGCTATCCAGGC	CTCCTTAATGTCACGCACGAT
	PTEN	TGGATTCGACTTAGACTTGACCT	GGTGGGTTATGGTCTTCAAAAGG
	DNMT1	CCTAGCCCCAGGATTACAAGG	ACTCATCCGATTTGGCTCTTTC
	DNMT3a	CCGATGCTGGGGACAAGAAT	CCCGTCATCCACCAAGACAC
	DNMT3b	CCCAGCTCTTACCTTACCATCG	GGTCCCCTATTCCAAACTCCT
	miR-23b-3p RT	CTCAACTGGTGTCGTGGAGTCGGCAATTCAGTTGAGGTAGTAAT	
	miR-23b-3p	ACACTCCAGCTGGGATCACATTGCCAGGGAT	TGGTGTCGTGGAGTCG
	U6	CTCGCTTCGGCAGCACA	AACGCTTCACGAATTTGCGT
mouse	E-cadherin	CAGTTCCGAGGTCTACACCTT	TGAATCGGGAGTCTTCCGAAAA
	N-cadherin	AGCGCAGTCTTACCGAAGG	TCGCTGCTTTCATACTGAACTTT
	Vimentin	CGTCCACACGCACCTACAG	GGGGGATGAGGAATAGAGGCT
	β-actin	GGCTGTATTCCCCTCCATCG	CCAGTTGGTAACAATGCCATGT
	PTEN	TGGATTCGACTTAGACTTGACCT	GCGGTGTCATAATGTCTCTCAG
	DNMT1	CCGTGGCTACGAGGAGAAC	TTGGGTTTCCGTTTAGTGGGG
	DNMT3a	GATGAGCCTGAGTATGAGGATGG	CAAGACACAATTCGGCCTGG
	DNMT3b	CGTTAATGGGAACTTCAGTGACC	CTGCGTGTAATTCAGAAGGCT
	miR-23b-3p RT	CTCAACTGGTGTCGTGGAGTCGGCAATTCAGTTGAGGGTAATCC	
	miR-23b-3p	ACACTCCAGCTGGGTGGGTTCCTGGCATGC	TGGTGTCGTGGAGTCG
	U6	CTCGCTTCGGCAGCACA	AACGCTTCACGAATTTGCGT
